# Supplementary figures and images for: BushenHuoxue formula promotes osteogenic differentiation via affecting Hedgehog signaling pathway in bone marrow stem cells to improve osteoporosis symptoms
Source: PLoS One. 2023 Nov 29;18(11):e0289912. doi: 10.1371/journal.pone.0289912 (PMC10686470; doi:10.1371/journal.pone.0289912)

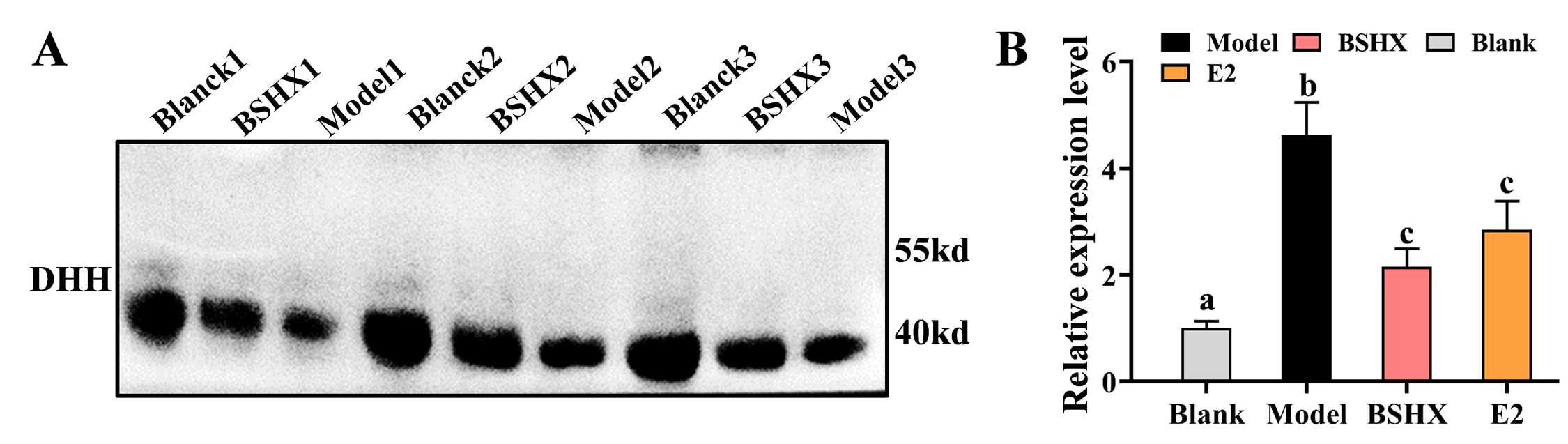

Supplement: S1 Fig — (TIF) [file pone.0289912.s001.tif]

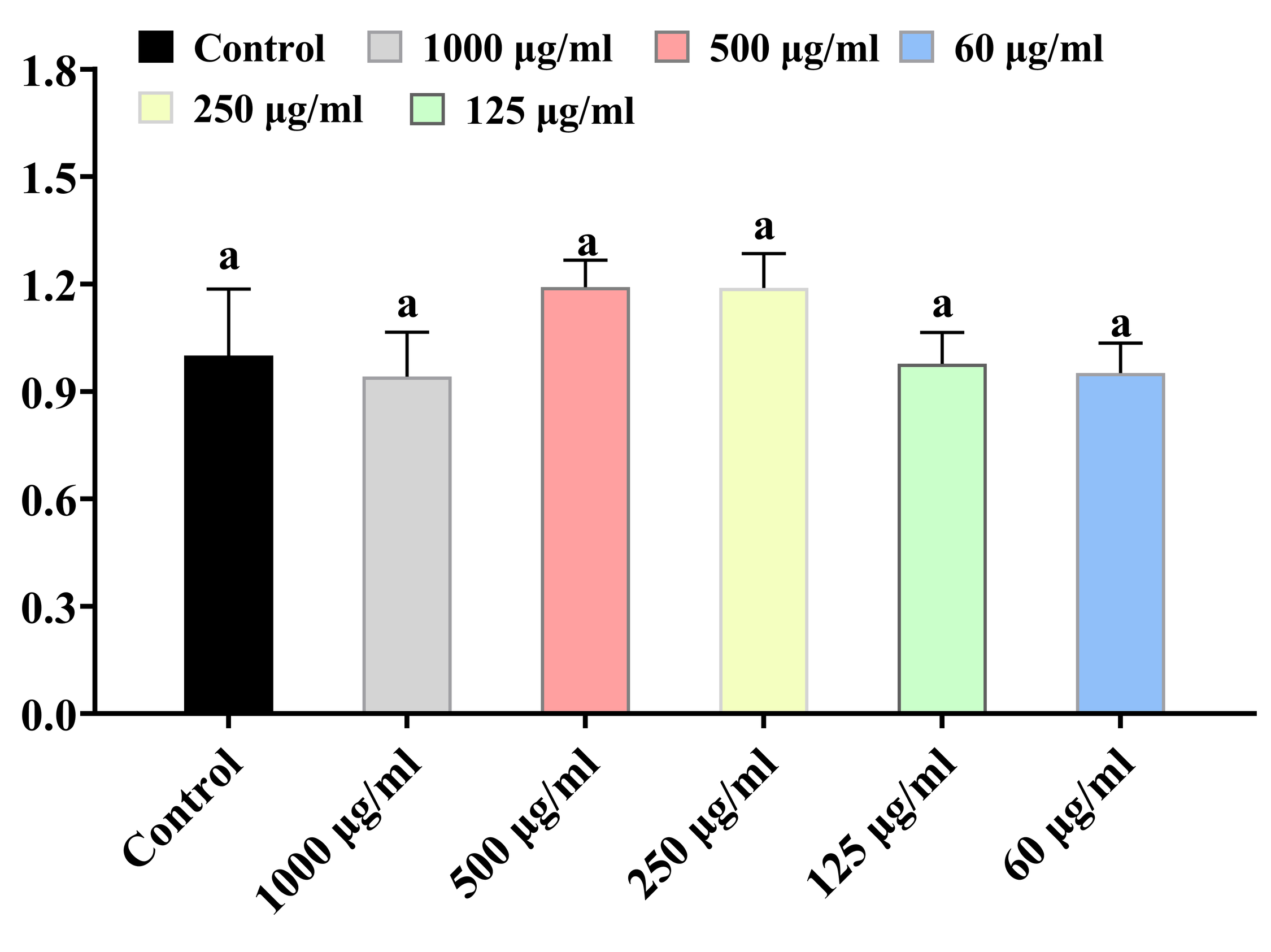

Supplement: S2 Fig — (TIF) [file pone.0289912.s002.tif]
